# Doctors that “doctor” sickness certificates: cunning intelligence as an ability and possibly a virtue among Swedish GPs

**DOI:** 10.1007/s11019-020-09954-3

**Published:** 2020-05-09

**Authors:** Mani Shutzberg

**Affiliations:** Centre for Studies in Practical Knowledge, Södertörn University, Stockholm, Sweden

**Keywords:** Metis, Cunning intelligence, Phronesis, Practical knowledge, Medical practice, Sickness certification

## Abstract

The relations of power between healthcare-related institutions and the professionals that interact with them are changing. Generally, the institutions are gaining the upper hand. Consequently, the intellectual abilities necessary for professionals to pursue the internal goods of healthcare are changing as well. A concrete case is the struggle over sickness benefits in Sweden, in which the *Swedish Social Insurance Agency* (SSIA) and physicians are important stakeholders. The SSIA has recently consolidated its power over the sickness certificates that doctors issue for their patients. The result has been a stricter gatekeeping of sickness benefits. In order to combat the inroads made by state institutions into sickness certification, and into the sphere of medical practice, some doctors have developed cunning “techniques” to maximize the chance to have their sickness certificates accepted by the SSIA. This article attempts to demonstrate that cunning intelligence—the ability of the weak to “outsmart” a stronger adversary—plays an important role in the practice of medicine. Cunning intelligence is not merely a defective form of prudence (*phronesis*), nor is it simply an instance of instrumental reason (*techne*), but rather an ability that occupies a distinct place among the intellectual abilities generally ascribed to professionals.

## Introduction

Aristotle’s *History of Animals* certainly demonstrates his knack for biology. However, with respect to the quote above, Aristotle was wrong. In addition to the cuttlefish, there is at least one more species that cunningly employs its ink for the sake of concealment: *the modern physician*. Recent observations indicate that members of this species write sickness certificates in a way that *appears* to comply with the stricter eligibility criteria for sickness benefits, but in order to evade them (Shutzberg 2019, 2020). This omission should not reflect badly on Aristotle. Healthcare and doctors were simply different in ancient times. Firstly, the use of ink for writing was not yet invented. Secondly, and probably much more important, the framework for medical practice was somewhat different back then.

Healthcare in late modernity is a different milieu, populated by other animals. Today, due to the rise of hospitals, welfare and management systems, massive healthcare enterprises etc., the multiplicity of stakeholders and actors in any given clinical situation modulates the clinical power of the physician. The last decades have borne witness to a negative modulation of clinical discretion, through audits and managerial techniques (Numerato et al. 2012; Power 1997). For better or for worse, the physician is less a subject of power, and more subjected to it. While oversight and control are not bad per se, the proliferation of goods and goals that are external to medical practice might misguide highly complex healthcare organizations.

As healthcare is different today, so too are the virtues ascribed to its practitioners. The practical situatedness and historical mutability of (professional) moral concepts and virtues certainly is not a new idea (see for example MacIntyre 2007, pp. 27–28; Pellegrino and Thomasma 1993, pp. 31–40). Cunning intelligence (or what the ancient Greeks referred to as *metis*), the ability of the weak to overcome a stronger adversary, makes its entrance as an important professional trait in a time when highly complex healthcare organizations are increasingly (mis)guided by external rather than the internal goods of healthcare.

Such is also the case in a facet of society that borders on healthcare, law and politics: social insurance, and the sickness benefit system in particular. The publicly financed social insurance system of Sweden has recently set off down the same path of austerity as insurance systems in many other welfare states. In an attempt to cut the costs of sickness absence, eligibility criteria for “disability” and “sickness” has narrowed, pressure to reintroduce unemployed “sick persons” into the labor market has intensified, and rigorous control mechanisms to monitor patients with approved sickness benefits has been put in place (Grover and Soldatic 2013; Burström 2015). The work and professional discretion of healthcare workers (mainly physicians), social service workers, and others involved with sickness and disability cases, are more tightly regulated than ever by performance indicators, scripts and routines, by organizational directives and governmental decrees (Broadbent and Laughlin 2002; Hasselbladh and Bejeroth 2017).

Physicians, however, do not seem to acquiesce to the reconfigured relations of power. They have demonstrated that the worn Foucauldian cliché—“where there is power, there is resistance”—holds at least some truth. Although weakened by the bureaucratic fabric of increasingly austere sickness benefit systems, doctors find discreet ways to navigate through them, in defense of their patients, their clinical autonomy, and to mitigate workload. In previously published qualitative studies, it has been shown that at least Swedish general practitioners (GPs) do so with “street-smart” stratagems, when writing sickness certificates to the *Swedish Social Insurance Agency*^1^ (SSIA; Shutzberg 2019). This street-smart comportment and set of “techniques” can be understood as a form of “everyday resistance” that functions through mounting an appearance of compliance (Shutzberg 2020). Hence, the less quoted subclause of the Foucauldian cliché is also true in this case: “[…] and yet, or rather consequently, this resistance is never in a position of exteriority in relation to power” (Foucault 1978, p. 95).

In this article I will explore the epistemological underpinnings of these “street-smart” stratagems. How does this bundle of intellectual ability, knowledge and practices employed in these situations relate to the traditional Aristotelian distinctions between the intellectual virtues? Is it warranted to introduce a new category? Can the category of cunning intelligence (*metis*)—well known among the Greeks but scorned by their philosophers—play a distinct but complementary role to the other “indispensable virtue for medicine”, namely prudence, or what the Greeks called *phronesis* (Pellegrino and Thomasma 1993, p. 90)?^2^

## Clinical judgment and prudence in sickness certification: the relation between the universal and particular

Maria is not feeling well. Neither sleep nor weekends help with recovering from the fatigue. Even minor physical and mental exertion leaves her drained for the rest of the day. Work has demanded a lot lately, and being available through e-mail around the clock does not help. She feels down. A perceptive colleague of her suggests that Maria visit her doctor. So she does. After a thorough check-up, her GP identifies the problem as a depressive episode and/or burnout syndrome. The GP determines that Maria’s ability to work is impaired. The observations, examination and judgment is written down on a form and submitted to the Swedish social insurance agency.^3^

The case above is a pretty routine case in Swedish primary care. The typical “difficult” sick listing case patient is usually presented as female, working in a socially demanding field (teacher, nurse, social worker, project manager, etc.), and suffering from a psychiatric condition (Shutzberg 2019; Engblom et al. 2008). In Sweden, patients who are unable to work due to medical conditions are economically compensated by the publicly financed sickness benefit system. Patient eligibility for sickness benefits requires that the treating doctor issues a sickness certificate, detailing the patient’s diagnosis, functional impairment (through observations, clinical examinations and tests) and the limitations to activities in relation to work. Based on the information in this standardized form, the SSIA makes the final call to approve or deny the patient’s claims (Riksrevisionen 2018, p. 22).

From the doctor’s point of view, insurance medicine and sickness certification has never been easy. All the general challenges of medical practice are relevant in this subset of medical practice as well. One is confronted by epistemological and ethical uncertainty within the four walls of the examination room that can be difficult and potentially dilemmatic to handle. Engblom et al. have proposed that most such difficulties, often fueled by conflicting interests between doctor and patient, fall within eight different categories: 1. Is the patient’s problem really medical in nature, and if not, will sick-listing solve the problem?; 2. Is the doctor the authority, or merely a servant yielding to the patient’s demand for sick-listing?; 3. What should the doctor do with discrepancies between what the patient says and the doctor finds?; 4. Should the doctor conduct exhaustive assessment of remaining working capacity that delays treatment and risks medicalization?; 5–6. May sick-listing itself cause (reversible or irreversible) harm?; 7. Should the doctor sick-list the patient despite personal doubts about its efficacy?; 8. May rehabilitative measures following sick-listing do more harm than good? (Engblom et al. 2008).

It is interesting to note that ethical and epistemological questions are tightly conflated in the difficulties and dilemmas enumerated above. Many things pertaining to the practice of medicine are not known, and many known things are imprecise: the ambiguity of what health means for this or that particular patient; the unpredictability of disease; the uncertainty of diagnosis and prognosis; the imprecision of diagnostic tools; the limits of human medical knowledge. In short, medicine is suffused with uncertainty on multiple levels—”moral, metaphysical and epistemic” (Tonelli and Upshur 2019, p. 507). Many of these general moral, metaphysical and epistemic challenges have been relatively constant since the inception of medicine as a profession.

Attempts to understand how doctors navigate in moral and epistemic uncertainty often make reference to *clinical judgment* (see for example Kienle and Kiene 2011; Montgomery 2005). As the literature in this field is too vast to deal with without some kind of selection, I will turn to a philosophical framework that has gained considerable traction in philosophy during the last 50 years, and in professional theory in general and medical theory in particular during the last 20 or so years: virtue ethics and Aristotelianism (Anscombe 1958; MacIntyre 2007; Pellegrino and Thomasma 1993; Banks and Gallagher 2009; Kinsella and Pitman 2012). In fact, Aristotle was one of the first to develop some kind of idea of judgment that tied together the moral and epistemic aspects of human action. From an Aristotelian point of view, “good clinical judgment is the end product of the use of prudence” (Pellegrino and Thomasma 1993, p. 90). What then is this “prudence” that Aristotle talks so highly of? As I see it, there are two essential dimensions to the intellectual virtue of prudence: 1. Prudence is the ability to bring together scientific and moral reasoning into a whole: “Anyone who has had to unravel the intricacies of clinical choices in a concrete case knows that the decision is the product of an intimate dialogue between the clinical facts and the moral principles, values, or virtues” (ibid: 90); 2. Prudence is at the same time a way of bringing the universal in dialogue with the particular case, such that the universal is put in the service of the particular (Aristotle 2011, p. 124 [1141b15-20]). Returning to the concrete case of Maria’s medical encounter can perhaps put things in perspective:

Maria’s GP knows that there are national guidelines for sick-listing patients. According to the so-called “insurance medical decisions support”^4^ provided for doctors by the *National Board of Health and Welfare*,^5^ sick-listing “mildly depressed” patients should be avoided. This certainly makes sense generally. But in Maria’s particular case, the fatigue seems to be partially caused by an unbearable workload. It is a difficult decision, as being off work could both exacerbate Maria’s condition, or help with recuperation. The experienced and prudent GP decides to make an exception in Maria’s particular case and sick-list her.

The way the GP acts in this situation is a mundane yet telling example of professional prudence. It is relevant in areas of clinical life where simple rule-following is inadequate. Someone truly capable in their professional field must be able to deliberate and act well in singular situations that lack both clear-cut universal guiding rules as well as identical precedents. It is so because the universals (laws, principles, precepts, etc.) never truly and exhaustively can say everything about the particulars.^6^ This is not to say that universals, principles, have no place in judgments. On the contrary, they give a necessary sense of general direction, a rule of thumb. Hence, it is *generally true* that depressed patients (such as Maria) are not helped by staying away from work. Yet, there may be particular circumstances that warrant sick-listing. Furthermore, that universals cannot give a full account of the to the particular case does not mean that rule-bound, productive and instrumental action has no place in medical practice.^7^ Quite the contrary. This logic of *techne* is operative on two distinct levels in cases such as Maria’s. Firstly, the *production of data* is precisely a *techne*: to interview the patient (perhaps with the aid of surveys), to physically examine her and order lab tests, and then to correctly translate those findings to text on the sickness certificate. Secondly, following existing guidelines and precepts in an instrumental manner is also a form of *techne*, insofar as the guideline is the universal which is mechanically translated onto the particular case. In Maria’s case, a doctor can look up the recommended duration of sick-listing in the national guidelines for a particular diagnosis. This way of passively relating to guidelines is perhaps more salient among less experienced practitioners. In complex cases, this instrumental relation to guidelines might lead to mistakes, but are nevertheless an important propaedeutic stage for developing proper prudence, i.e. an ability to act wisely in the particular case.^8^ Empirically this also seems to hold true, as less experienced non-specialists report more appreciation for the “*insurance medical decisions support”* (Svärd et al. 2018, p. 2).

## New challenges to sickness certification practice: age of bureaucratic austerity and the limits of prudence

The importance of prudence (in the Aristotelian sense) in sickness certification matters has not changed dramatically over time. The metaphysical relationship between the universal and the particular, that is, the challenges of *applying* what one knows and the principles one holds on the particular and unique case is principally the same today as it was twenty years ago. Clinical prudence still demands years of experience to perfect.

At the same time, the institutional setting of sickness certification in Sweden has changed dramatically since the early 2000s; these changes, mainly the reconfiguration of the relations of power between physicians and the institutions, have had far-reaching consequences for the exercise of prudence. Once, the role of the SSIA in determining patient eligibility for sickness benefits was less prominent. The doctor was the main de facto “gatekeeper*”* of access to sickness benefits (Sundquist et al. 2007). Due to ideological and political pressure, the power over sickness benefits has gradually shifted from the physicians to the government and the SSIA. In the 1990s the Swedish public discourse on how work disability was being problematized started to shift: it went from being mainly a public health concern to concern with patients’ unwillingness to work, with the implication that the latter reflects misuse of social benefit systems (Johnson 2010). Doctors working in different jurisdictions, not only in Sweden, were accused of aiding in this alleged overuse of sickness benefits by passively yielding to patient demand (Arrelöv, Edlund and Goine 2006; Hussey et al. 2004). Consequently, on the level of public policies, changes came creeping from the late 2000s and onwards. For example, the government stipulated an upper limit to the duration of sickness benefit eligibility in 2008 (which has since been partially rolled back), effectively pushing the boundary between medicine and law towards the latter. Since then, the SSIA review process of sickness certificates have gradually become more rigorous (Larsson et al. 2017). In summary, the SSIA has established itself as the *gatekeeper of the gatekeepers*.

Nationwide surveys have shown that doctors, especially GPs, are not pleased with these reforms. Between 2004 and 2017, the proportion of Swedish GPs who reported that their medical assessments were questioned by the SSIA spiked from 10 to 57%. The number of GPs who experienced that the SSIA requested unnecessary corrections to the sickness certificates increased from 48 to 72% between 2012 and 2017. In 2017, 72% of GPs believed that the SSIA requested that doctors provide “objective signs” of illness in their sickness certificates in cases where such objective signs are difficult to identify (e.g. psychiatric disability, chronic pain, etc.) (Alexanderson et al. 2018). Finally, a very important and telling complaint has been reported by the national surveys with doctors: “doctors perceive that the SSIA does not implement the *insurance medical decision support* as guiding recommendations, but as ‘laws’” (Svärd et al. 2018, p. 2). The so-called *insurance medical decision support* that was implemented on a national level to help guide doctors in their sickness certification practices, have been hijacked by the SSIA to exert power over the behavior of doctors.

When guides turn into imperative lawlike structures in this way, the relationship between prudence and the universal is altered. This new situation reveals one conceptual limit to Aristotle’s concept of prudence. Aristotle seems convinced that the prudent have the choice to accept or reject certain laws and precepts. Or put differently, if the essence of prudence is to decide on making exceptions to the rule, then the freedom to make exceptions must be assumed. In his discussion of justice and equity in the *Nicomachean Ethics*, for example, Aristotle notes that the prudent needs to rectify what the law necessarily misses, because the generality of law cannot capture particular circumstances (Aristotle 2011, p. 112 [1137b10-b35]).^9^ This may very well be the case for the sovereign in power—but not so much for the ruled who are in a non-negotiable relationship with the law. Aristotle reiterates the intimate connection between prudence and power by presenting the powerful statesman Pericles as *the* role model of prudence in the sixth book of the *Nicomachean Ethics* (Aristotle 2011, pp. 120–121 [1140b5-b15]). When Aristotle claims that “political art and prudence are the same characteristic”, he understands politics from the point of view of rulers (Aristotle 2011, p. 124 [1141b20-b25]). In *Politics*, Aristotle even goes so far as to say that while many virtues can be common between rulers and those ruled, prudence is “the only virtue peculiar to a ruler” (Aristotle 1998, p. 73 [1277b25-30]).^10^

Hence, although it is reasonable to claim that doctors need to be prudent in relation to their patients, over which they “rule” to some extent, it is problematic to say that the relationship between doctors and institutions in this age of bureaucracy is adequately understood through the lens of prudence. This is because doctors are “ruled” by institutions more than they are their rulers. It is also important to note that the relationship between universals and particulars is not only solely a metaphysical issue. It is at the same time political, in the sense that the universal (in the form of standardized guidelines, rules, laws, precepts, etc.) may force itself on the particular cases to varying degrees. In sickness certification matters, the universal throws a dark shadow on the particular patient cases, and the degree of freedom of the individual medical practitioner to prudently establish a dialogue between them is smaller than before. The challenge for doctors is, then, not how to rule well but rather, how *not* to be ruled. How *not* to be ruled requires another intellectual capacity altogether, to which I will now turn.

## Beyond the examination room: cunning intelligence in sickness certification

The GP tells Maria that he needs to be “street-smart” when issuing the certificate. Sure, Maria can still laugh occasionally, and her affects are not completely blunted. Yet, the doctor tells Maria that it is better to actually write that her affects are blunted, in order to match the “ideal” depressed patient. The doctor explains that the Swedish Social Insurance Agency has been ordered by the government to keep down the total number of granted sick days. Even patients with serious disabilities are deemed unqualified for sickness benefits, sometimes due to some minor detail in the sickness certificate. In order to circumvent additional paperwork, defend the autonomy of medical practice, and last but not least, to guarantee adequate treatment for the patient (in this case, Maria), her doctor “doctors” the sickness certificate.

The nationwide Swedish survey in which doctors reported that the SSIA use the national guidelines for sickness certification (the so-called *insurance medical decision support*) as an instrument of control over physicians. However, the report also hinted at the occurrence of an opposite phenomenon: Physicians admitted to using the insurance medical decision support in an “unintended way, for example as a basis for finding a diagnosis whose recommendations better correlate to the duration of sick-listing that the doctor deems adequate for the medical condition of the patient” (Svärd et al. 2018, p. 2). These doctors seem to *reverse engineer* the guidelines-turned-laws in order to use them against the SSIA themselves. It is not unreasonable to assume that these voluntary reports of unintended use of the guidelines is only the top of an iceberg of evasive actions.

One of the central criteria for sickness benefit eligibility that has caused many headaches for Swedish physicians and their patients is the way the SSIA understand the concept of “objective signs” of disease. In many cases of disease that affect the ability to work, there are no lab tests or radiological exams to independently verify the condition. Such is the case with psychiatric conditions, chronic pain, etc. In these cases, SSIA’s increasingly narrow interpretation of “objective signs” has led to a higher degree of rejected sickness benefit claims, counter to the clinical judgments of the treating physicians. Because of this, GPs have developed a cluster of “techniques”, that is, “informal, and unsanctioned, ways of maximizing the likelihood of sickness certificate acceptance by the SSIA.” In an interview study with Swedish GPs on this matter, the doctors themselves labeled them expressions of “street-smartness”, “tactics”, “maneuvering around” (Shutzberg 2019). The common denominator of these “techniques” is that they in one way or another deal with the SSIA’s inflexible and narrow understanding of “objective signs” when deciding eligibility for sickness benefits. Some techniques aim at (1) producing *quasi-objectivity*: for example, GPs reported that the SSIA tended to dismiss patients’ own accounts since it narrowed the eligibility criteria, as patient accounts do not qualify as “objective signs” that can be independently verifiable. Yet, in many medical conditions that limit the activity level of patients, what the patient says is all there is. Psychiatric conditions, chronic pain etc. *can* manifest themselves in the examination room, but not necessarily. Hence, the doctors reported that they would write the sickness certificate such that the patient’s narrative appeared as an “objective finding” made in the examination room. A narrative account of suicidal thoughts, lowered mood and so on, could be written as something the doctor encountered in the consulting room (e.g. “patient appears with a lowered mood”, rather that “the patient *says* that she usually suffers from lowered mood”). Other “techniques” aimed at (2) *evading objectivity*: for example, when SSIA requested supplemental information, GPs could phone SSIA case workers and “communicate off the record”. In these conversations, doctors conveyed information that could not fit in the reified format of the standardized sickness certificate form, such as feelings, intuitions etc. about their patients’ medical conditions. Sometimes the physicians could use other means of persuasion, such as pointing out their superior medical knowledge relative to the SSIA case workers. Thirdly, a few techniques (3) *produced objectivity at a price*: In order to satisfy the narrow view of “objective findings”, they would be produced despite being medically unnecessary. For example, patients with conditions such as chronic back pain would be submitted to extra radiological examinations, although such examination would not alter the treatment strategies in any way.^11^

Not unlike Aristotle’s cunning cuttlefish, doctors employ these techniques in order to *conceal* their resistance to the strict eligibility criteria enforced by the SSIA. They break the iron bars of bureaucracy precisely by pretending to follow them. But *what* are these new set of techniques that doctors utilize in order to navigate in a complex and increasingly inflexible bureaucracy? Are they just modifications of the same old prudence displayed by the seasoned doctor in relation to his or her patients? Or are they something else, epistemologically and ethically? Perhaps the Greeks, who astutely theorized prudence, did have something to say about the shrewdness of such a mode of thought and action as well?

## Steps towards providing cunning intelligence its own space among the intellectual abilities

Despite Aristotle’s fascination with the cunning cuttlefish, he scorned that very same ability in the life of humans. In discussing prudence in the sixth book of the *Nicomachean ethics*, Aristotle made the passing remark that “[t]here is indeed a capacity that people call ‘cleverness,’ and this is of such a character as to be capable of doing what is conducive to the target posited and so of hitting it. If, then, the target is a noble one, the cleverness is praiseworthy; but if base, it is mere *cunning* [*panourgia*]” (Aristotle 2011, p. 131 [1144a25-30], my italics). Although there is not much in this passage to interpret, it does at least hint at the existence of *something* similar to, yet distinct from, prudence in cunningness. Aristotle settles for giving cunningness a privative definition (it is similar to prudence, but without an understanding of what is good and truly virtuous) and leaves it at that. This tendency of reducing ruse and trickery to something resembling prudence, but in the end being nothing more than rule-bound and instrumental “binding”, “trapping”, “controlling” and “immobilizing” of instrumental knowledge (*techne*) is also to some degree repeated among the neo-Aristotelians of our days (see for example Dunne 1993, pp. 256–58; Nussbaum 2001, pp. 20, 310).

Yet, outside of philosophy, in the universe of myth, the Greeks did indeed demonstrate a deep appreciation for cunning intelligence. Marcel Detienne and Jean-Pierre Vernant’s meticulous work on cunning intelligence in Greek myth, *Cunning Intelligence in Greek Culture and Society*, is an ambitious attempt to reconstruct the Greek appreciation of this intellectual capacity—*metis*—from the myths of the Gods. More a “semantic field” than a unified concept, it encompasses everything from navigating ships across shifty seas, to gods outsmarting other gods. Given the vast amount of different examples of *metis* provided by Detienne and Vernant, a broad interpretation of metis would evidently have it overlapping with the practical intellectual capacities and virtues of Aristotle, with *techne* and *phronesis*, also in a very literal sense: Both *techne* and *phronesis* are are at different points in the book fused together with the category of *metis* (Detienne and Vernant 1991, pp. 43, 138).

However, some fundamental characteristics ascribed to *metis* by Detienne and Vernant seems to warrant its presentation as a *particular sub-species* of practical knowledge, somewhat distinct from both productive instrumental knowledge (*techne*) and practical-ethical knowledge (*phronesis*). If one attempts to isolate this aspect of *metis*, five tightly interrelated features stand out as unique, or at least helpful for contrastive purposes. *Firstly*, metis comes in play in antagonistic circumstances, in a race or contest, a conflict, a rivalry or the like. The first example of *metis* presented by Detienne and Vernant is the chariot race recounted in the 23rd book of the *Iliad*, which I will return to below. *Secondly*, more specifically, metis is crucial for the subjugated part in this antagonistic conflict. One could even claim that metis is almost exclusively the weapon of the weak, circumventing the advantage of pure strength of a more powerful adversary. Instead of pure strength, *metis* is “the use of methods of a different order whose effect is, precisely, to reverse the natural outcome of the encounter and to allow victory to fall to the party whose defeat had appeared inevitable” (Detienne and Vernant 1991, p. 13). In the chariot race, Antilochus beats Menelaus to the finish line despite having slower horses. He does so through taking “advantage of a sudden narrowing of the track, which has been worn away by storm rains, and drives his chariot obliquely across in front of that of Menelaus at the risk of causing a crash: the manoeuvre takes his adversary by surprise and he is forced to rein in his horses” (Detienne and Vernant 1991, p. 12). *Thirdly*, cunning intelligence is that form of thought and movement brought about by being completely interior to power. What I mean by this is that metis becomes necessary when there is nowhere outside of the terrain of power to run or hide, and when the setting of the potential confrontation between antagonistic forces is structurally “rigged” and set. The myth of Metis—wife of Zeus and mother of Athena—is itself a metaphor of the particular *type* of subjugation that necessitates *metis*: In order to cheat his own fate which was to be overthrown by his own future son, Zeus swallows Metis who is pregnant with their child (1991, p. 60). Metis is, then, literally *inside* of rule and power. This aspect of cunning intelligence is perhaps more obvious in the comportment of the trickster god Prometheus, the most cunning of all. “Hesiod and Aeschylus are at one in recognising in Prometheus that very type of wiley intelligence, that same power of deception which the Greeks called *metis*” (1991, p. 58). Detienne and Vernant highlight the myth of how Prometheus outwitted Zeus when a huge ox was to be slaughtered and divided between the humans and the gods. Prometheus divided the ox into two portions for Zeus to pick from, knowing that gods have the first pick. “The first looks extremely appetising but in reality consists only of the animal's bare bones. The second conceals beneath the skin and stomach—which are inedible—all the best pieces” (1991, pp. 125–126). Zeus of course picks the first, getting only bones, while the humans were allotted the edible parts. As the hierarchical order between humans and gods were set, as well as the order in which the meat was distributed, Prometheus had to trick Zeus. However, it was a trick that on a superficial level still “respects” the order of things. Being completely swallowed up by power also means that the subjugated must seize whatever tools and opportunities that the situation or the master presents. Doing so successfully is the essence cunning. In the case of Prometheus, it was the picking order that was cunningly exploited. *Fourthly*, deception and disguise are indispensable components of metis. “In metis appearance and reality no longer correspond to one another but stand in contrast, producing an effect of illusion, *apate*, which beguiles the adversary into error and leaves” (Detienne and Vernant 1991, p. 21). In addition to this fourth feature, it follows—*fifthly*—that metis is first and foremost a *social* skill. This fifth interpersonal and social dimension is not unique for *metis*. *Phronesis*, being intimately related to action (*praxis*), is an activity between humans, which has been emphasized by for example Hannah Arendt (1998), but also by Phillippe Baumard, who calls *phronesis* “social wisdom” (1999, p. 53). However, this fifth aspect does to some degree distinguish *metis* from *techne,* which expresses itself in the *fabrication* of things (*poiesis*), that is, as a relation between a subject and an object, rather than *acting* in relation with others.

That the ancient Greeks did demonstrate appreciation for the capacity of cunning does not automatically make it a virtue. Nevertheless, the *ontologically* positive exposition of cunning intelligence gives the phenomenon substance, instead of reducing it to the mere shadow or defective version of some other human capacity (such as in Aristotle’s case, prudence).

## *Metis* in the age of bureaucracy: resistance of practice and practice of resistance

At first glance, the concept of *metis* seems very distant from practice in the world of modern organizations and institutions. Yet, the concept has been mobilized by several thinkers in order to make sense of practice within and against bureaucracy. Among them, political scientist and anthropologist James C. Scott has been the most empirically oriented in attempts to shed light on the complicated interplay between cunning intelligence and institutions.^12^ In his highly influential work *Seeing Like a State,* Scott asks why authoritarian high-modernist projects (such as scientific forestry in the late eighteenth century, forced agricultural collectivization during the Soviet era, centralized urban planning projects influenced by Le Corbusier in the mid twentieth century, etc.) often ended in terrible failures. The argument is that these projects are destined to simplify reality in the name of efficiency and control and are therefore, by design, incapable of appreciating the particularity of “*this* forest, *this* revolution, *this* farm” (Scott 1998, p. 318). This extends to an inability on behalf of the bureaucratic state structure to recognize particular and local forms of tacit and approximative knowledge of local farmers, artisans, physicians and what have you. Taking Detienne and Vernant’s work as a point of departure, Scott adopts the Greek word *metis* to designate this dimension of practical knowledge inherent (albeit to different degrees) in all human activities, ranging from riding a bicycle and local farming to diplomacy, politics, and just about everything in between. His explicit thesis is that failure of high-modernist projects is due to the fact that *metis* “*resists* simplification into deductive principles which can be successfully transmitted through book learning, because the environments in which it is exercised are so complex and nonrepeatable that formal procedures of rational decision making are impossible to apply” (Scott 1998, pp. 315–316, my italics). The explicit thesis is, then, that practical knowledge *passively* resists due to its ontological refusal to be codified. It is simply impossible to codify highly contextualized and particular forms of practical knowledge.

This broad take on practical knowledge and the resistance it engenders, certainly overlaps with established concepts such as “practical knowledge”, “procedural knowledge”, (Gilbert Ryle’s) “knowing how”, (Michael Polanyi’s) “tacit knowledge”, (Aristotle’s) “*phronesis*”, or any of the other alternatives mentioned by Scott himself (“indigenous technical knowledge”, “folk wisdom”, “practical skills”). All these concepts—irrespective of the radical differences between them—denote knowledge that to some degree requires context and person, and that refuse complete codification or conversion to propositional knowledge. Their total subjugation to centralized systems is, almost by definition, epistemologically impossible. What Scott says of the passive resistance of *metis*, is also the case with practical knowledge in many of the varieties above. By their very nature, they all passively “resist simplification” and reduction to “mechanical application of generic rules” (1998, p. 318).

An alternative reading of some of the central examples Scott provides the reader is possible, with the implication that there is more to *metis* than the mere passive irreducibility, survival and resistance of practical knowledge itself. This alternative reading focuses on the *kind of professional practices whose intended objective is resistance against institutions*, rather than the resistance that passively follows from the exercise of professional practice. So, for example, Scott mentions a study of how East German factory workers dealt with meeting the production quotas set by the central government. As access to necessary spare parts and raw materials were scarce and unreliable, some of the workers also acted as dealers on the black market:

the factory routinely used its funds to stock up on such valued nonperishable goods as soap powder, cosmetics, [...] and fashionable clothes. When it seemed that the plant would fall short of the quota [...], these knowledgeable dealers would set off across the country [...], to secure what was needed. Neither of these roles was provided for in the official table of organization, and yet the survival of the factory depended more on their skills, wisdom, and experience than on those of any other employee. A key element in the centrally planned economy was underwritten, always unofficially, by metis. (Scott 1998: pp. 350-351)

The shrewdness of the workers moonlighting as dealers in this case is not a simple return to old ways of producing goods, which would be impossible. Neither is it the passive resistance of older forms of practical knowledge that have managed to survive incorporation into centralized planning. Rather, the quota system and its unavoidable imperfections gives rise to a higher-order and a new form of knowledge and practice that aims at circumventing the quota system itself. It has few or no links to old ways farming, woodcutting or mining, although it might at times function as a bulwark against their extermination. The relation between this cunning intelligence and the institutions it acts against involves a series of contradictions: it simultaneously *resists and consolidates* the centralized systems, which simultaneously *produces and represses* these types of disposition, knowledge and action.

Let’s recall the trick at Mecone, mentioned earlier, for a second. Prometheus tricked Zeus into picking the pile of bones, leaving the edible flesh of the ox for the humans. But who tricked whom, really? Vernant suggests that Hesiod’s *Theogony* can be interpreted such that the ultimate trickster was Zeus himself, letting Prometheus believe he was tricked, while in fact he was not. Zeus wanted the bones of the ox, the substrate of immortality. He wanted a reason to condemn humans to mortality, and Prometheus gave him an opportunity to do so by his insubordination (Detienne and Vernant 1989, pp 224–225, n. 6). The same potential inversion of who profits (in the long run) from the cunning intelligence of employees in organizations is the implicit theme of Scott’s case, and relevant in the case of cunning GPs. Whom does it really serve that GPs cunningly outsmart the SSIA by subtle means, by creating an apparent compliance with the new stricter rules of sick-listing? Is it possible that the resistance of *metis* is an inadvertent but life-sustaining feature of the sickness certification system?

## Synthesis and concretion: *phronesis* and *metis* as complementary abilities in sickness certification practice

Let’s return to the sickness certificates. When issuing sickness certificates, two complementary modes of knowledge are mobilized by the GP. Firstly, in terms of the content, *this* particular patient before the GP calls for a professional judgment to be made. Is sick leave beneficial for this particular patient? How much, in what way? Are other medical measures necessary? These kinds of judgments exist in the realm of *phronesis*. They are fundamentally *unmanageable*, in the sense that they refuse submission to universal categories. Or put differently: when universal categories are confronted by a particular case, judgment is needed and cannot be wholly supplanted by flowcharts and algorithms. Still, the sphere of medical judgment is continuously encroached by external goods (such as economic efficiency) and control mechanisms (such as governmental decrees and guidelines, as well as extensive auditing of the sickness certificates), seeking to minimize professional discretion. The greater the encroachment, the more GPs grow another different and distinct sphere of knowledge and ability: *metis*. As understood in the previous section, this is the ability to create a space for judgment and action through disguise. This ability is partially independent of the knowledge, ability and virtue of prudence (*phronesis*) in relation to the patient. That is, it is easy to imagine a doctor who is prudent in relation to his or her patients, but incompetent with regards to outsmarting social insurance agencies or other forms of bureaucracy (when it is legitimate to do so). Yet, these two abilities (of *phronesis* and *metis*) are indirectly related. The “function” of *metis* is to keep fundamentally unmanageable space unmanaged. It is closely related to prudence, but different in kind. While *phronesis* deals with the content of a unique situation that must be handled wisely, *metis* creates the conditions under which that unique situation *can* be dealt with wisely. While *phronesis* may be called the “tricks of the trade (in a nondeceptive sense)”, only metaphorically as Scott did (1998, p. 330), *metis* is the “tricks of the trade” in the highest possible literal sense of the phrase. Doctoring is an essential component of being a doctor.

Hence, *phronesis* and *metis* differ from each other with regards to their area of interest: *phronesis* deals with the clinical content, while *metis* deals with (some aspects) of the clinical context. They also differ with how they relate to rule. Grossly simplified *techne*, or instrumental knowledge, is the ability to follow rules; *phronesis*, or prudence, is the ability to act where the domain of rules end; *metis*, or cunning intelligence, is the ability to act in between, or against, rules. Consequently, they differ in their relation to uncertainty: While prudence deals with the ontological uncertainty of disease itself, with prognosis and treatment, *metis* deals with the organizational uncertainty of a bureaucratic system that tends to wipe out the particularities of *this* or *that* patient.

## The ambiguous ethical and political status of *metis*: Why lumping *phronesis* and *metis* together is a bad idea

Many scholars of medical philosophy have identified the challenge of acting well in a bureaucratic structure in medical practice. A common position for the scholars in the field influenced by Aristotle’s intellectual virtues is to subsume the creatively evasive skills involved in sickness certification under the category of prudence (*phronesis*). Saraga et al., for example, identify both rule-breaking in general (and “gaming the clinical context” in particular), as well as “creating the magic bubble” (i.e. a temporary space, shielding the clinical encounter from external interference) as essential aspects of “*phronesis* enacted in medicine” (Saraga et al. 2019, p. 49). In a similar vein, Bontemps-Hommen et al. arrive at the conclusion that the definition of professional prudence needs to be updated to account for the complexity of “late modernity”. Consequently, their proposed updated definition of *phronesis* reads as follows:

practical wisdom [prudence] is the capability which emerges in acting jointly in medical practices, consisting of knowing how to remain focused on achieving the good for every individual patient, within the context of the practice and its telos, in ever changing situations, and how to accomplish this through the most appropriate means, *while operating complexity and institutional and systemic pressure.* (Bontemps-Hommen et al. 2019: p. 103, my italics)

The last subordinate clause (italicized above) is of central interest here: “operating […] institutional and systemic pressure” is identified not as distinct from, but as a part of professional prudence in late modernity. Both formal and informal, both rule-following, rule-bending and rule-breaking ways of dealing with this pressure is implicitly incorporated in this refusal to differentiate between different ways of dealing with institutional and systemic pressure.

The importance of identifying the novel challenges of “late modernity,” “bureaucracy,” “institutions” etc. to good doctoring and the *skills* they require cannot be overstated. Both Bontemps-Hommen et al. and Saraga et al. have successfully done so. However, highlighting their importance by elevating them to the realm of prudence (*phronesis*) in the Aristotelian sense, risks obfuscating some essential differences between prudence and the cunning ability to maneuver in bureaucracies. Most importantly, some of these differences are related to the ethical and political status of *phronesis* and *metis*. They emanate from three central characteristics of metis: its *oppositionality*, its *secrecy* and its *enactment from subordination*. While phronesis to some extent always can be institutionally recognized and sanctioned as an important part of professional activity, metis cannot. At best, *metis* can be institutionally *tolerated* (as acknowledged by James C. Scott) but never accepted. Consequently, attempts can be made to deliberately and publicly facilitate the learning of phronesis, but because metis in its very essence is the ability to counter institutions, it cannot enjoy the same privilege. Metis can be taught horizontally only, whispered between colleagues, but never vertically, as part of a public curriculum.

## The million-dollar question: Is *metis* a virtue?

I have hitherto talked about *metis* as form of knowledge and/or capacity pertaining to the physician’s professional role. In this regard, *metis* is similar to *phronesis*, which also can be understood as a kind of (ethico-practical) knowledge possessed by doctors, and/or as a kind of capacity belonging to that professional role. But prudence is not merely an attribute like any other—it is a *virtue.* The attempts at differentiating *metis* from *phronesis* above may boil down to one question that is simple to ask, but difficult to answer: Is *metis* a virtue? It would certainly be easy to dismiss cunning intelligence as a defective mode of *phronesis*, as Aristotle did, or dismiss it on the basis that it collides with Alasdair MacIntyre’s idea that honesty is one of (at least) three central virtues of modern human practices.^13^ Is cunning intelligence not simply the intellectual capacity for dishonesty, the necessity of which might be excused because of pressing circumstances, but never acclaimed as a virtue? In order to answer this question, I believe a turn to MacIntyre’s neo-Aristotelian framework (in the form it has been presented in *After Virtue*) can be helpful, as he has expanded on the relevance of virtues in modern society in which human practices are both supported and suppressed by institutions (such as a “hospital” in which the practice of medicine takes place, which MacIntyre himself uses as an example). MacIntyre defines virtue in the following way:

A virtue is an acquired human quality the possession and exercise of which tends to enable us to achieve those goods which are internal to practices and the lack of which effectively prevents us from achieving any such goods. (MacIntyre 2007: p. 191)

This (preliminary) definition of virtue brings its relation to “practice” to the foreground, which in this context has a semi-technical meaning, close to, but slightly different from the broader colloquial use of the word. A practice is.

any coherent and complex form of socially established cooperative human activity through which goods internal to that form of activity are realized in the course of trying to achieve those standards of excellence which are appropriate to, and partially definitive of, that form of activity (MacIntyre 2007: p. 187).

Again, MacIntyre is helpful by giving examples of practices: medicine is a practice (MacIntyre 2007, p. 194). The good internal to the practice of medicine has been considered to be health, which is the essential goal and reward of this practice. However, there are other goods contingently tied to the practice of medicine, that are external to it: fame, status, money, etc. The dual nature of goods, as it were, are also applicable in the case of sickness benefits: on the one hand sickness benefits are something to be used with the health of the patient in mind. Or better yet, medically informed sickness certification practice manages to put external goods (economic compensation) in the service of health. Unfortunately, on the other hand, the opposite relationship between the inner good of health and the external good (of bureaucratic efficiency) is not only thinkable, but a rule in contemporary Swedish social security arrangements. Narrow and strict eligibility criteria for sickness benefits have turned social security from an aid to health to a tool for forcing sick patients back to the labor market.

To repeat the chain of reasoning of this article, but with a MacIntyrean vocabulary, and in terms of virtue rather than intellectual capacity, *metis*, then, is a virtue, because “without them [the virtues], practices could not resist the corrupting power of institutions” (MacIntyre 2007, p. 194). *Metis*, the cunning aspect of medical practice, is that which guarantees that sickness certification practice (which is a part of medical practice) operates in accordance with the inner good of health, rather than the external good of efficiency and austerity. It is the covert way to “resist” the external goods of institutions gone adrift.

The preliminary answer to the question is then: yes, *metis* is a virtue. However, there are some serious reservations to be made. Firstly, Macintyre’s idea of virtue is much more complex and relates not only to practice. In addition to practice, virtues are tied to a good life as a whole (which may be hard to reconcile with the idea of *exclusively* professional virtues); furthermore, virtues are intimately connected to the inheritance of a living tradition. A second type of reservation to calling *metis* a virtue has been raised by other philosophers. The neo-Aristotelian philosopher Lisa Tessman, who has contributed to understanding virtues under non-ideal conditions and making virtue ethics relevant for emancipatory projects, has noted that there are some important differences between traits that are virtues both in ideal and non-ideal circumstances, and traits that “deserve praise only in the qualified sense of being the best that is possible under awful conditions” (Tessman 2005, p. 7). Clearly, *phronesis* belongs to the first category, and *metis* to the second. If one chooses to call the second category of traits “virtues”, it should be with the qualification that they are “burdened” or “burdening” virtues, Tessman suggests.

For these reasons, a *definitive* answer to the question of whether *metis* is a virtue should be postponed for future study.

## Conclusions

This paper has argued that the recently changed relations of power between medical practitioners and the SSIA has forced Swedish GPs to develop a new type of ability. This ability, cunning intelligence, is sufficiently different from technical instrumental rationality (*techne*) and prudence (*phronesis*) and therefore deserves its own category: *metis*. There are reasons to assume that this intellectual ability is generalizable. Firstly, the concept has a far-reaching history, delineated by Detienne and Vernant, and by James C. Scott. Secondly, the factors that necessitated *metis* are not limited to the Swedish social insurance system. Rather, the logic of austerity and the bureaucratic control over professionals have become paradigmatic in healthcare (Siegler 1985; Power 1997).

Prudence is considered to be “[m]edicine’s indispensable virtue” (Pellegrino and Thomasma 1993, p. 84) because those who bear it are able to take into consideration which other virtues, which ends and goals are relevant in situations fraught with uncertainty. Prudent conduct of physicians in the domain of sick-listing is certainly a challenge in itself: what benefits *this* particular patient? In the setting of bureaucratic austerity, there is the additional obstacle to effectuate these judgments. The ability to cunningly circumvent the heteronomy of rules and regulations, the external goods of institutions that force themselves on the sphere of medical practice, is a distinct intellectual capacity. It is a capacity necessitated by subordination.

The unique place of *metis* in the repertoire of intellectual capacities can be expressed in its relation to rules: instrumental reason is the ability to *follow rules*; prudence is the ability to move and act *where rules do not apply*; cunning intelligence is the ability to move and act *despite rules*. Cunning complements prudence.

Cunning intelligence is more than mere defective prudence. Nevertheless, the ontologically positive status of cunning intelligence does not automatically make it a virtue. This investigation paves the way for asking whether it might be. A preliminary answer is that cunning intelligence perhaps is a virtue, or at least that it is tightly interwoven with professional virtues and the internal goods of healthcare. A definitive answer to this question lies in the future.

## Footnotes

1 Official translation of the Swedish government agency “Försäkringskassan”.

2 In this article, *prudence* is used as the English equivalent of the Greek concept of *phronesis.* Another common translation is *(practical) wisdom*. Opting for prudence rather than wisdom is purely arbitrary.

3 The case of “Maria” is fictional, but based on examples of difficult sick-listing cases recounted by Swedish GPs in an interview study (Shutzberg 2019).

4 Unofficial translation of the Swedish “Försäkringsmedicinskt beslutsstöd”.

5 Official translation of the Swedish government agency “Socialstyrelsen”.

6 “And prudence [*phronesis*] is not concerned with the universals alone but must also be acquainted with the particulars: it is bound up with action, and action concerns the particulars” (Aristotle 2011, p. 124 [1141b15-20]).

7 To what extent “*techne*” itself is knowledge of the particularity of a situation, or closer to the universal, has been debated. On one end stands for example Bent Flyvbjerg, who sees in *techne* a focus on “that which is variable and particular”, similar to prudence (2008: p. 492). On the other hand there is the almost opposite tendency, in for example Joseph Dunne, to emphasize its proximity to *episteme* (1993, p. 251) and with reference to Aristotelians such as Aquinas as something that proceeds “according to fixed and classical methods which is the rule of *ars* rather than of *prudentia*” (Aquinas, quoted by Dunne 1993, p. 253). Dunne also explicitly takes *techne* to be the antithesis of the particular: “once we descend to particular cases of dealing with *this* or *this*, we are no longer securely within the governance of the *techne*, which always remains limited to *general* rules” (Dunne 1993, p. 259). Devereux also equates *techne* with the universal (1986, p. 493). In this article I will stick to this second interpretation of *techne.*

8 Hubert and Stuart Dreyfus famously articulated the acquisition of skill as necessarily beginning in rule-following and progressing toward something akin to prudence (Dreyfus and Dreyfus 1986, pp. 16–51).

9 Although Aristotle does not explicitly mention prudence in conjunction with his discussion on equity and justice in this passage, I will follow Martha Nussbaum and Daniel Devereux in recognizing the proximity to prudence (Nussbaum 2001, p. 301; Devereux 1986, p. 496).

10 Bent Flyvbjerg has also pointed out this lack of problematization of power in Aristotle’s conception of phronesis, simply because it “may not have been a problem for practising *phronesis* among the elite in the Greek *polis*” (2008, p. 504). Furthermore, Richard Bernstein confronts Aristotle by proxy through a critical engagement with Gadamer concerning his omission of power when discussing *phronesis* (Bernstein 1983, pp. 157–159). Although both Flyvberg and Bernstein make very important points in this regard, neither directly address (political) power as the presupposed vantage point of *phronesis*.

11 For a more detailed and empirical account of these “techniques”, previously published, see Shutzberg (2019). For an account of the use of the techniques as a form of everyday resistance, and a closer look at the intent and motives of resisting physicians, see Shutzberg (2020).

12 Philippe Baumard (1999) and Michel de Certeau (1984) are two others who pursued a similar interest in the concept of *metis* (as it has been understood by Detienne and Vernant) and its relation to organization, institution and bureaucracy. For the sake of clarity and brevity, I will stick to James C. Scott’s more empirical engagement with the concept.

13 The other two being justice and courage (MacIntyre 2007, p. 191).
